# Distortions in Spatial Mental Representation Affect Sketch Maps in Young Adults

**DOI:** 10.3390/neurolint14040064

**Published:** 2022-09-21

**Authors:** Antonella Lopez, Andrea Bosco

**Affiliations:** 1 Faculty of Law, Giustino Fortunato University, 82100 Benevento, Italy; 2 Department of Educational Sciences, Psychology, Communication, University of Bari, 70121 Bari, Italy

**Keywords:** cognitive distortion, sketch maps, familiar environment, psychometrics

## Abstract

Humans tend to misrepresent spatial information which leads to systematic errors due to distorted organizational processes regarding metric and positional judgments. This study is aimed at analyzing metric and positional distortions in cognitive maps by using external representations, namely sketch maps, in two experiments with young participants. In the first experiment, we use the sketching area of Northern Europe. In the second experiment, the University campus area was used. The first aim was to test the hypothesis that the presence of the sea between the triplet of landmarks generates an overestimation of the distances between them in the case of Northern Europe; and to test the hypothesis that the number of turns in a route influences the overestimation of the distance between landmarks in the case of the campus area. The second aim was to investigate alignment and rotation errors using the same maps. Concerning metric errors, the results showed the overestimation of distances with a geographical gap between the cities (the sea in the Northern European Area), and those with more turns between landmarks (the campus area). The results concerning rotations and alignments were in line with the previous research about positional errors. The present study extended findings on distortions in spatial mental representation as emerging from verbal judgments, to sketch maps: direct visuospatial external representations eluding the conversion into verbal coding of spatial information. The presence of distortions in cognitive maps could be considered the consequence of the encoding of spatial information.

## 1. Introduction

The human perception of spatial arrangements plays tricks on our minds. When we are convinced that what we are thinking about (e.g., spatial configurations and/or large-scale space) is the faithful copy of reality, that is what the trap of the mental representation reveals. During the processes of perception, learning, and storing of spatial information, some of it is misrepresented, leading to systematic errors in the representation of the environment. First of all, gestalt principles explain how humans organize individual elements into groups and how humans perceive and recognize patterns. For the purpose of this study, the more relevant principles are proximity, for which elements close to each other are grouped together, or the principle of common region used to perceive depth relations after some depth perception process has been completed [1,2]. Moreover, as stated by McDonald and Pellegrino [3], the human system of spatial representation tends to function in an efficient and economic manner from the point of view of the resources deployed. Making detailed and accurate representations could become very expensive for human spatial cognitive encoding. This phenomenon could be considered a sort of cognitive shortcut, maybe aimed at not overloading our memory. This failure of representing spatial information could be considered a normal process regarding memory for spatial environments, namely cognitive distortions [4,5].

### 1.1. Representation of Spatial Information in Humans

Cognitive maps contain information about the location, position, distances, and direction between objects, with a share of incorporated systematic patterns of distortions e.g., alignment and/or rotation [4]. Moreover, as shown by Tversky [6], humans misrepresent spatial information, leading to systematic errors due to a distorted organizational process regarding metric and positional distortions. Humans usually make judgments about distance and direction using a hierarchical organization of the environment [7]. In other words, humans use a hierarchical encoding of spatial information, referring to a superordinate category that guides their decisions (e.g., distance judgments: the state in which the cities are located) [8,9]. As an example of this, the findings of Maki [10] showed an increase in speed of the response time, judging distance and direction for intercluster locations (i.e., regions, nations) compared to intracluster locations, demonstrating the presence of a superordinate—hierarchically higher—level of processing with which people create cognitive maps. Therefore, it is reasonable to state that the hierarchical organization of cognitive maps is a central property of spatial memory. Humans create their own spatial hierarchies even if no physical or perceptual boundaries are present. In this regard, McNamara et al. [11] (p. 225) stated that “*These structures determine psychological distances between remembered locations and in ways that often do not correspond to Euclidean distances between actual locations*”. Using a recognition priming task, they showed a personal hierarchy of the participants, having learned about the location of the objects on a small-scale spatial configuration, and the organizing of objects into “chunks” or clusters. Likewise, in the mental representation of the space at an ecological level, people need mechanisms for organizing their memories into units consistent with the limitations of their memory systems.

### 1.2. Distortions in Cognitive Maps

Regarding metric distortions, when people were requested to estimate distances between points of reference, they overestimated across-cluster distances; conversely, they underestimated in-cluster distances. Firstly, humans acquire the location of landmarks, and then the other characteristics of the environment. Landmarks often become points of reference that induce a violation of the true distance relations, producing distance asymmetries [4]. The effect of reference points can produce asymmetrical distance judgments [12]. Typically, distances between cities closer to the reference point were inflated with respect to distances between cities farther from it. Lloyd and Heivly [13] showed the tendency to overestimate shorter distances more than longer distances in aggregate urban cognitive maps. Other findings have shown the inconsistency in representations of distance, as in the case of Holyoak and Mah [12], who asked participants to estimate how far east cities were from the Pacific coast, and how far west cities were from the Atlantic coast. The participants overestimated the distances in both directions. In both cases, the authors described this effect as an enlargement of space near a reference point so that distances to near places were overstated and distances to places far from the reference point were reduced. Moreover, the presence of geographical barriers and environmental features was investigated by different researchers with the same results: distances across barriers, such as rivers, hills, railroad tracks, and buildings, are overestimated in contrast to comparable distances that do not cross a barrier [14,15,16,17,18]. Briggs [19] showed that familiarity with landmarks along urban routes influenced distance judgments for those routes. Other researchers have verified that the number of turns in a route is decisive for estimating distance: routes with more turns were evaluated as longer than those with fewer turns [20,21,22]. The reason for this finding was explained by Allen and Kirasic [23], who showed that humans have the tendency to subdivide routes into segments, and then use the segment boundaries as markers to estimate distance. Supporting these results, Thorndyke [24] also found that routes with more turns were estimated as longer than those with fewer turns. Moreover, McNamara and et al. [11] found that distance estimates were determined more by route distance (City Block) than by Euclidean distance; therefore, the number of turns was important in the distance evaluation.

Regarding positional distortions, alignment and rotation errors are present. Alignment error is a phenomenon of alteration of direction due to a grouping by proximity: landmarks are perceived clustered together but skewed, namely, more aligned with respect to the north/south or east/west axis than they really are. Tversky [6] revealed a tendency to represent South America as being aligned with the south of North America, when in fact South America is eastward. Similar results were reported by Muller [25] and Lloyd and Heivly [13] regarding the San Francisco Bay and the city of Columbia in South Carolina, respectively. Tversky [6] argued that humans tend to simplify the spatial mental representations encoding landmarks in straight lines, even when this is not true. Rotation errors regard a conflict between the orientation of the actual landmarks compared to the orientation observed in the participant’s representation. They are like alignment errors: the individual translates or tilts the position of landmarks to the vertical or horizontal side of the frame [26]. The findings present in the literature are a little bit tricky and controversial; in fact, some researchers e.g., [27] have reported both types of error for configuration acquired through navigation and map study, and others have suggested more errors due to alignment and rotation for representations acquired from primary learning with respect to secondary learning e.g., [28]. Direction errors regard distortion in the representation of directions [29] and the biases produced in the estimation of the location of the reference points. Findings showed that, generally, angles were biased towards 90°, suggesting a fundamental inconsistency in the represented directions. Furthermore, as shown by Friedman and Brown [30] the location estimates of cities did not depend on item knowledge, but instead, were based on regional prototypes or some other sort of general knowledge about the regions. They examined this question by comparing the latitudes and longitudes between cities in North America and Europe, and the results showed more distortions in between-region cities than within-region cities.

Nevertheless, what makes these findings controversial are the experimental procedures. It is argued that the distortions in spatial mental representations partly depend on the procedure/task used. Huttenlocher et al. [31] introduced the category adjustment model (CAM), which posits that participants imperfectly remember stimuli in serial judgment tasks. In order to maximize accuracy, CAM holds that participants use information about the distribution of the stimuli to improve their judgments. CAM predicts that judgments will be a weighted average of imperfect memories of the stimuli and the mean of the distribution of stimuli. More interestingly, in the abovementioned studies, results come from tasks in which participants retrieve spatial information in the form of verbal judgments. This kind of task requires primarily visual information to be translated into a verbal/propositional format, and verbal judgments entail effects due to verbal transcoding, e.g., verbal overshadowing [32]. It may be possible to state that distortions in cognitive maps could be, at least partially, the consequence of the detrimental effects of verbalizing non-verbal information. As shown by Fiore and Schooler [33] there could be a reactive effect of verbal reports on spatial mental models, and in fact verbalization hindered performance on a measure of configural knowledge (straight-line distance estimations). It was shown that participants who were forced to verbalize various processes performed more poorly on certain tasks or made less satisfactory decisions than participants who did not perform any type of verbalization [34,35,36,37].

### 1.3. The Present Study

So, given the consolidated results in the literature regarding spatial distortions in verbal judgments, the present study is devoted to investigating metric and positional distortions in cognitive maps, analyzing a simplified version of the sketch map as external representations of spatial information acquired through primary and secondary learning. Sketch maps graphically represent the environment, drawing it on a sheet of paper, placing certain objects in a specific location, and thinking about the spatial configuration from a bird’s eye view or along a route. Thus, sketch maps, as the externalization of cognitive maps, the internalized reflection, and the reconstruction of space in thought [38], reproduce schematizations that originate in cognitive maps [39]. Sketch maps can be considered a reliable method to externalize and represent collected spatial information [3,40].

Then, using two landmark location tasks, a simplified version of sketch maps composed of only three landmarks, e.g., [41,42], regarding spatial information, we wanted to evaluate which kind of distortions were produced, and if the findings already shown in the previous research, about spatial verbal judgments, could be extended to graphic representations of the environment, a format to convey information that does not require the conversion from a visuospatial to a verbal–spatial coding. This kind of task has already been used in a series of studies e.g., [7,41,42], in which the authors reported information about the selection of the landmarks, based on a pilot aimed at rating participants’ level of knowledge and familiarity with landmarks. 

The first aim was to investigate metric distortions. Using the geographic area of Northern Europe (Experiment 1), we wanted to test the hypothesis that the presence of a boundary such as a sea between a triplet of landmarks (Paris, London, and Amsterdam) generated an overestimation of the distances between them [16,18]. Moreover, using the area of the University of Bari campus (Experiment 2), we wanted to test the hypothesis that the number of turns in a route influenced the overestimation of the distance between three landmarks (the entrance to the Student Center, the entrance of the Department, and the stairs of Salone Affreschi).

Furthermore, a second aim in both experiments was to investigate alignment and rotation errors using the same areas. We wanted to explore if both types of errors were present, suggesting that such errors represent fundamental patterns of distortion in cognitive maps.

## 2. Materials and Methods

### 2.1. Experiment 1

The first experiment aimed at investigating metric distortions on the external representation of a geographic area of Northern Europe. In the first instance, Experiment 1 aimed at verifying distance errors in a group of young participants performing the task. Second, it aimed at assessing alignment and rotation errors in the same task. Specific assumptions could be made on the influence of the presence of the sea between the points of reference chosen, inducing participants to overestimate the distance between them. No specific assumption could be made for alignment and rotation errors.

#### 2.1.1. Participants

Two hundred and sixty participants (131 women) took part in the study. All participants were from the metropolitan area of Bari, Apulia, Italy. They were young Italian university students who responded to an advertisement without compensation. All participants, blind to the hypothesis of the study, signed a consensus form. The participants were enrolled between December 2016 and April 2018. The whole sample was admitted into the assessment to investigate their ability to retrieve spatial information previously learnt during their life experience mainly through map study or incidentally. The Ethics Committee of the Institution approved the study protocol (n. 3660-CEL03/17), and the whole study was performed following the Helsinki Declaration and its later amendments.

The inclusion criterion for young participants was academic performance considered as a measure of cognitive efficacy [41,42,43,44,45,46]. Young participants had high/adequate academic achievement measured as the number of exams per year (inclusion cut-off: five or more exams; maximum number of exams per year: seven). They had a general level of familiarity with the geographical area investigated, rating them on four items: the use of Google Maps, paper maps, weather forecasts, and the study of geography, on a scale from 1 (=never) to 7 (=always). Means and standard deviations for all the criteria for inclusion are reported in Table 1.

#### 2.1.2. Materials and Procedure

**Figure 1:**
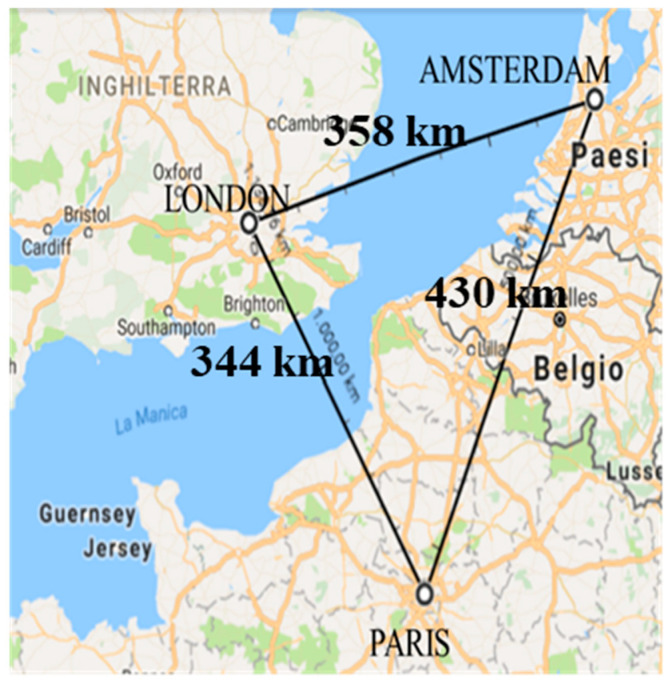
Northern European Area and distances between landmarks. Illustration from Google Maps (https://maps.google.com, accessed on 6 September 2022).

Afterward, participants were required to carry out the Northern European Area estimation which included three landmarks: Paris, London, and Amsterdam. The area investigated was approximately 63,012 km^2^ (see Figure 1). The stated scale was 1 cm = 162 km. The way in which participants had learned the entire area was more likely through map study. The triplet was characterized by the largest distance between the two points of reference placed on the mainland: Paris and Amsterdam. The medium distance was between London and Amsterdam. The minimum distance was between London and Paris. Both distances have the sea as a natural barrier between the cities.

We used an empty “sketching area” oriented in portrait format, measuring 11.3 cm × 12 cm e.g., [47], facing north. Participants only had to pinpoint the landmarks for each geographical area, respectively, keeping in mind metric (i.e., relative distances) as well as categorical (“A is North/South and East/West is B”) spatial relations between landmarks (see Figure 1). The participants responded to the following instructions: “Think of the spatial relationships between the landmarks. In the box below, draw three crosses, corresponding to the landmarks, and label them. Please use the full sketching area. Please be careful to respect the proportional distances between landmarks and their correct positions relative to each other”.

The entire procedure was made clear to the participants beforehand. They were assessed individually in a well-lit and quiet room without disturbances. Data were collected in one session. The whole assessment lasted a maximum of 5 min.

#### 2.1.3. Statistical Analysis

Data were analyzed using R as statistical software (version 4.2.1, Vienna, Austria). The cleaning up and the structuring of the data was performed using Microsoft Excel software. The Northern European Area was composed of three distance estimations different from one another. Firstly, the analysis of distance errors was conducted starting from the measurement of the Cartesian coordinate of each landmark. Then, the distance between landmarks was calculated using the sum of the absolute differences of Cartesian coordinates between two points in the plane i.e., Manhattan Distance [41]. Finally, distances were transformed into rank order data. Rank order data have been used in the past to study the effects of cognitive distortions e.g., [14,16].

Two kinds of errors are expected: (a) the shortest distance (London–Paris) being represented as the medium or the largest, and (b) the medium distance (London–Amsterdam) being represented as the largest. Both cases can be conceived in terms of an overestimation of distances due to the effect of the gap represented by the Channel Strait.

In order to analyze ordinal level data from a repeated measurement experimental design, the Friedman two-way analysis of variance by ranks test and the multiple comparisons procedure using the Wilcoxon matched pairs test were performed. Under the null hypothesis, we assumed no effect between sets of scores (London–Paris, Lon-don–Amsterdam, Paris–Amsterdam) alternatively, one set of scores differed from another. We calculated Cohen’s r effect size for a Wilcoxon signed rank test by dividing the test statistics by the square root of the number of observations e.g., [48].

In order to investigate positional errors (alignments and rotations), we evaluated the slope of the line going through each couple of landmarks. To a smaller angle inclination, compared with the correct one, corresponded the tendency to align; conversely, to a larger slope corresponded the propensity to rotate. Also, in this case, we transformed the slopes into rank order data, and we performed Friedman’s two-way analysis of variance by ranks test and the multiple comparisons procedure using the Wilcoxon matched pairs test. For each couple of landmarks of the triplet, participants had: (a) the possibility to estimate exactly the position of each couple of landmarks (correct response); (b) the option to align the position of each couple of landmarks; and (c) to rotate the position of each couple of landmarks.

### 2.2. Experiment 2

The second experiment aimed at investigating the presence of distance errors, and alignment and rotation errors in performing a landmark location task based on the University of Bari campus area. Specific assumptions could be made about the presence of more turns. Distances with more turns should be estimated as longer than those with fewer turns. Again, no specific assumption could be made for alignment and rotation errors.

#### 2.2.1. Participants

Two hundred participants (102 women) took part in the study. As in Experiment 1 they were students at the University of Bari, coming from the metropolitan area of Bari, Apulia, Italy.

#### 2.2.2. Materials and Procedure

**Figure 2:**
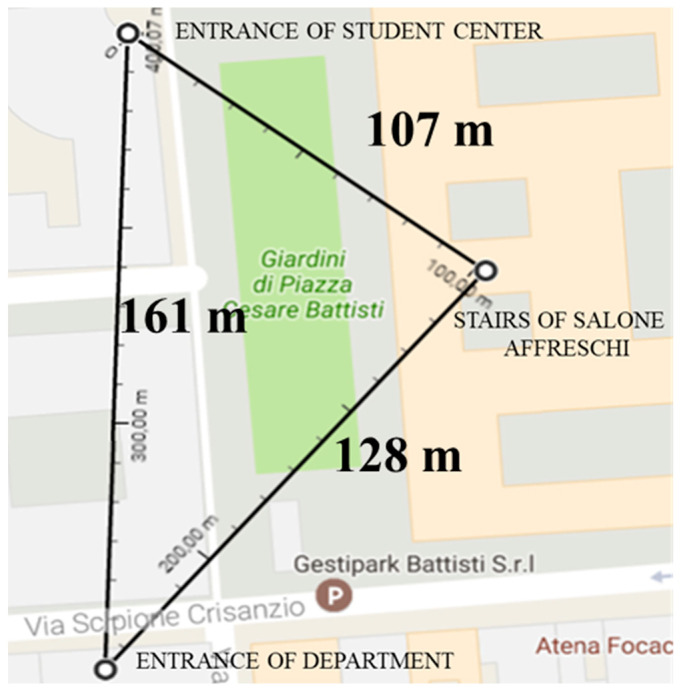
Campus area of the University of Bari and distances between landmarks. Illustration from Google Maps (https://maps.google.com, accessed on 6 September 2022).

The setting, procedure, and inclusion criteria were the same as in Experiment 1. Participants were required to carry out the map of the University of Bari campus. It included three very familiar and memorable landmarks of the area of the campus: the entrance to the Student Center, the entrance of the Department, and the stairs of Salone Affreschi inside the main building of the University. The walkable area of the campus was approximately 6.6 km^2^ (see Figure 2). The stated scale (relationship between distances on a map and distances in real life) was 1 cm = 19 m. The way in which the participants learned the entire area was mainly through repeated navigation experiences. The campus of the University of Bari was characterized by three salient landmarks of the university citadel classified by more turns for each distance. The walkable area between the Student Center entrance and the Department entrance was a straight line. The other two distances included one turn for the Student Center entrance–stairs of Salone Affreschi and two turns for the Department entrance–stairs of Salone Affreschi.

#### 2.2.3. Statistical Analysis

Data were analyzed using the same statistical approach as in Experiment 1.

## 3. Results

### 3.1. Experiment 1

Descriptive statistics for the participants (i.e., age M ± SD = 23.38 ± 2.72; year of education M ± SD = 15.17 ± 0.94) are reported in Table 1.

To accomplish the first purpose of Experiment 1 regarding distance evaluation, the Friedman statistic, which is calculated from the sums of ranks and the sample sizes, for the Northern European Area (distances: London–Paris, London–Amsterdam, and Paris–Amsterdam) was significant (FR(2) = 54,127, *p* < 0.001), showing a variability that affected the sums of the ranks: at least one distance differs from the others.

The Wilcoxon matched pairs test revealed that both the comparisons of London–Paris (sum of ranks = 568) and London–Amsterdam (sum of ranks = 568) with Paris–Amsterdam (sum of ranks = 424) showed a statistical difference (z = 6.98, *p* < 0.001 and z = 6.39, *p* < 0.001), with a medium effects size (r = 0.31 and r = 0.30 respectively), evidencing the overestimation of the minor and the medium distances probably characterized by the presence of the gap of the sea compared to the distance between cities on the mainland (Paris–Amsterdam). No difference (z = 0.09, *p* = 0.93) emerged in terms of sum of ranks between the London–Paris (568) and London–Amsterdam (568) distances.

The second aim of Experiment 1 concerned the evaluation of positional errors. The value of Friedman’s statistic was statistically significant (FR(2) = 513.39, *p* < 0.001). Performing the Wilcoxon matched pairs test, no differences emerged from the comparison between the sum of ranks of London–Paris (522) and London–Amsterdam (520) positions (z = 1.73, *p* = 0.25), showing, in both cases, the participants’ tendency to rotate the position of the cities (see Table 2). A strong tendency to align Paris and Amsterdam (778) emerged from the inspection of the comparison between the ranks of London–Paris and Paris–Amsterdam, and London–Amsterdam and Paris–Amsterdam (z = −16, *p* < 0.001, r = 0.70; z = −16.1, *p* < 0.001, r = 0.70, respectively, see Table 3).

### 3.2. Experiment 2

Descriptive statistics for participants (i.e., age M ± SD = 22.87 ± 2.77; year of education M ± SD = 15.17 ± 0.94) are reported in Table 4.

The University of Bari campus area was composed of three distances, characterized by the presence of a greater number of turns. As in Experiment 1, the evaluation of distance carries the possibility of overestimating and underestimating the distances or giving the correct judgments. From the Friedman two-way analysis of variance by ranks test, emerged a significant result (FR(2) = 9.07, *p* < 0.05). Again, variability affected the sums of the ranks, for every distance (the Student Center entrance–the Department entrance, the Student Center entrance–stairs of Salone Affreschi, and the Department entrance–stairs of Salone Affreschi). Moreover, the Wilcoxon matched pairs test revealed a significant difference between the sum of ranks of the Student Center entrance–stairs of Salone Affreschi (sum of ranks = 396) from the Department entrance–stairs of Salone Affreschi (sum of ranks = 431) distance (z = −2.06, *p* < 0.05), with a small effects size (r = 0.10 showing an effect of overestimation of the distance between the Department entrance and the stairs of Salone Affreschi, with more turns compared to the other. The difference in terms of sum of ranks between the Student Center entrance–the stairs of Salone Affreschi and the Student Center entrance–Department entrance (sum of ranks = 373) was revealed to be not significant (z = 1.61, *p* = 0.107); conversely, the difference in terms of the sum of ranks between the Department entrance–stairs of Salone Affreschi and the Student Center entrance–Department entrance, showed a significant difference (z = 2.41, *p* < 0.05), with a small effects size (r = 0.12), evidencing the underestimation of the distance with the absence of turns.

In the evaluation of positional errors, a significant result was obtained using Friedman’s statistics (FR(2) = 186.4, *p* < 0.001, see Table 5). The Wilcoxon matched pairs test showed the tendency to rotate the position of the Student Center entrance and the stairs of Salone Affreschi (468, z = −9.33, *p* < 0.001, r = 0.45) with respect to the Department entrance and the stairs of Salone Affreschi (572, z = −10.51, *p* < 0.001, r = 0.52), and the position of the Student Center entrance and the Department entrance (591, z = −3.54, *p* < 0.001, r = 0.15), that are lined up (see Table 6).

## 4. Discussion

The core aim of the present study was to analyze the presence of systematic distortions in spatial mental representations emerging from sketch maps. Humans create mental images of the environment in which they live, learned through navigation and other symbolic substitutes, namely, spatial sources, such as map study and verbal descriptions. Until the work of Ekman and Bratfisch [49], in which they studied the effect of subjective perception and emotions on distance estimation, it was thought that humans accurately represented distances and directions between landmarks, e.g., [18,50]. Then a series of studies were conducted on cognitive distortions. The novelty of the present study was to examine the distortions (e.g., distance errors and positional errors) in cognitive maps using external representations of spatial information: a simplified version of the sketch map, and a landmark location task. The way in which participants had to complete the task was by depicting landmarks enhancing a modality-specific visual encoding e.g., [51], and avoiding introducing a potential source of distortion: verbal conversion of spatial information stored in the memory, i.e., verbal overshadowing [32].

In Experiment 1, participants were asked to represent three cities of the Northern European Area. Starting from the evaluation of distance errors, and according to previous findings [14,16], we hypothesized an effect of overestimation related to distances containing the gap of the sea. The results showed the effect of overestimation of London–Paris and London–Amsterdam, with respect to Paris–Amsterdam that, consequently, was underestimated. This evidence is in line with previous findings on the effects of geographical obstacles e.g., [16]. Generally, there was an effect of overestimation due to the presence of a barrier, in our study a stretch of sea; conversely, distances that did not cross a barrier were perceived as smaller than they are. As shown in the work of Thorndyke [24], this result was in line with the hierarchical model of representation of space in which systematic errors occurred in making directional judgments, generating a superordinate relationship between landmarks. Therefore, the presence of the sea created a superordinate cluster, bringing an overestimation of the distance between landmarks.

Moreover, from a more qualitative point of view, both rotations and alignment errors emerged. It was interesting to notice a greater tendency to rotate the positions of the cities separated by the sea, and conversely, a propensity to align Amsterdam and Paris located on the mainland. It is difficult to explain this result with general theories, but we can still resort to hierarchical encoding. We were able to define these kinds of distortions as relative errors e.g., [8,28] due to the accuracy with which individuals encoded for certain locations. A person could have accurate and complete information about higher-order locations, such as countries, but less accurate information about the location of cities. Besides, the participants’ position estimations suffered from a further bias due to the “general superposition of the states” [18] (p. 749) to which the cities belonged.

We dare to say that the tendency to align Paris and Amsterdam, or to rotate the position of London, was probably due to the introjected perception of Northern Europe in a two-dimensional image. In order to represent the sphere of the Earth, cartographers transformed the Earth into a flat plane, distorting the relative sizes and positions of the continents. Individuals have an inaccurate view of Northern Europe, having a misleading perception of alignments between cities, even if they are on the mainland [52].

In any case, whether it concerns distance errors or more qualitative categorical errors, our evidence was corroborated by medium and large effect sizes, which upholds all these conclusions.

So, we agree with those researchers e.g., [53] who assumed that the acquisition of this kind of spatial knowledge probably influenced in some ways the encoding of spatial information, inducing some biases in constructing spatial representations. We know that spatial learning processes are complex and multifaceted, especially with map-like views acquired far back in time, and we had no possibility to experimentally control the acquisition of spatial information. We can only verify ex post how such perceptual/mnemonic biases influenced the mental transformation of representations.

In Experiment 2, we used the University of Bari campus area. The result was in line with the literature mainly regarding verbal judgments, e.g., [20,21,22], showing an overestimation of distances with the presence of more turns. The environment acquired through extensive behaviors of exploration and navigation, requires having learnt spatial information in strictly horizontal and/or vertical path walking. Consequently, the metric acquisition of the environment changed from a Euclidean to a Manhattan [54] distance type. The Euclidean distance can be defined as a straight line between two points, and the Manhattan or City block distance can be defined as the distance between two points, and it is the sum of the (absolute) differences of their coordinates. From the point of view of spatial navigation, humans move from one place to another by physical action. When there is an obstacle between two objects, it is impossible to pass through it, and humans must bypass it in order to find a way around it. Humans possess knowledge of how to get from one place to another. This knowledge regards the mental representation of directions and distances.

This interpretation brings us to estimate the length of the shortest path between two points as zero if the two points are really the same point, and greater than zero if they are distinct points, and the length of any path from one landmark to another that passes through some other buildings (e.g., city blocks). It might be longer, but it will never be shorter than the reality. These assumptions supported our findings with encouraging evidence: the distances with more turns were perceived as being longer than those without obstacles, notwithstanding it was the longest. However, the possibility that travel duration could have an effect on distance estimation cannot be excluded.

An interesting result came from the analysis of rotation and alignment errors. It seemed that participants had the tendency to align the position of landmarks (Department entrance and stairs of Salone Affreschi), Our results are in line with previous research, e.g., [28].

The present study has some limitations. Firstly, the study does not present a within condition considering how people would verbally present the landmark distances and positions. So, our conclusions are cautious. This kind of condition will be implemented in the future. Furthermore, not having used a metric approach for the statistics, information about the relative differences between object pairs was lost. Moreover, the portion of space referring to the Northern European Area is considered a large-scale environment compared to the small walkable area of the University campus. This difference is not passable. Therefore, the kind of geographical barriers were different: the presence of the sea in the Northern European area and the presence of buildings in the campus area. Further investigations will be conducted using more stimuli in a new experiment, for a more in-depth discussion of the differences between the two types of boundaries, and why such differences did not lead to different results. Finally, we did not have a value of specific self-reported familiarity for each landmark, specifically regarding the travel experience within the areas.

## 5. Conclusions

The present study represents another small piece to add to the line of research regarding biases in mental representations employing sketch maps. From the present study it emerges that distortions in cognitive maps (i.e., both metric and positional errors) arise independently of the kind of task. Using a simple landmark location task, asking to simply depict positions of a set of landmarks, the results seem to be in line with the existing literature. Unfortunately, it is not possible to evaluate cognitive maps directly, as expected by McDonald and Pellegrino [3]. The use of sketching areas is the nearest attempt to do this, but the evaluation is always indirect. The presence of distortions in cognitive maps could be considered the direct effect of the encoding of spatial information. Retrieving information in a verbal or visual format seems not change the kinds of distortions produced.

## Figures and Tables

**Table 1:** Descriptive statistics of the sample. Means ± standard deviations for interval and frequencies for nominal variables were reported. χ^2^ for frequencies were performed.

Variables	Experiment 1 (*n* = 260)
Gender, F/M	131/129
Age, years	23.38 ± 2.72
Education, years	15.17 ± 0.94
Academic performance	5.75 ± 0.8
*Familiarity with Northern Europe*	
Study of Geography at school	4.66 ± 2.08
Google Maps	6.11 ± 0.83
Paper Maps	2.84 ± 1.70
Weather Forecast	5.29 ± 1.60

**Table 2:** For distance errors and positional errors in the Northern European Area, the total sample size, *n*, Friedman chi-square, χ^2^, the degrees of freedom for Friedman chi-square, df; the *p*-value, *p*, were reported.

**Northern Europe**	***n***	**Distance Errors**			**Positional Errors**		
		**Friedman χ^2^**	**df**	***p***	**Friedman χ^2^**	**df**	***p***
	260	54,127	2	<0.001	513.39	2	<0.001

**Table 3:** For distance errors and positional errors in the Northern European Area, the sum of ranks, *n*, z score, the *p*-value, *p*, and the Cohen’s r of the Wilcoxon matched pairs test were reported.

Northern Europe	Sum of Ranks	Distance Errors			Sum of Ranks	Positional Errors		
		z	*p*	*r*		z	*p*	*r*
London–Paris/London–Amsterdam	568	0.092	0.93	-	522	1.73	0.25	-
London–Paris/Paris–Amsterdam	568	6.98	<0.001	0.31	520	−16	<0.001	0.70
London–Amsterdam/Paris–Amsterdam	424	6.39	<0.001	0.30	778	−16.1	<0.001	0.70

**Table 4:** Descriptive statistics of the sample. Means ± standard deviations for interval and frequencies for nominal variables were reported. χ^2^ for frequencies were performed.

Variables	Experiment 2 (*n* = 200)
Gender, F/M	102/98
Age, years	22.87 ± 2.77
Education, years	15.64 ± 1.31
Academic performance	5.99 ± 0.53
*Familiarity with Campus*	
Study of Geography at school	4.58 ± 1.79
Google Maps	5.68 ± 1.67
Paper Maps	3.55 ± 1.87
Weather Forecast	5.09 ± 1.86

**Table 5:** For distance errors and positional errors of the campus area, total sample size, *n*, Friedman chi-square, χ^2^, the degrees of freedom for Friedman chi-square, df; the *p*-value, *p*, were reported.

**Campus**	***n***	**Distance Errors**			**Positional Errors**		
		**Friedman χ^2^**	**df**	***p***	**Friedman χ^2^**	**df**	***p***
	200	9.07	2	0.001	186.4	2	<0.001

**Table 6:** For distance errors and positional errors of the campus area, the sum of ranks, *n*, z score, the *p*-value, *p*, and Cohen’s r of the Wilcoxon matched pairs test were reported.

Campus	Sum of Ranks	Distance Errors			Sum of Ranks	Positional Errors		
		z	*p*	*r*		z	*p*	*r*
Stairs–Student Centre/Stairs–Department	396	−2.06	0.05	0.10	468	−9.33	<0.001	0.45
Stairs–Student Centre/Department–Student Centre	431	1.61	0.11	-	572	−10.51	<0.001	0.52
Stairs–Department/Department–Student Centre	373	2.41	0.04	0.12	591	−3.54	<0.001	0.15

## Data Availability

The data presented in this study are available on request from the corresponding author.

## References

[1] Palmer S.E. (1992). Common region: A new principle of perceptual grouping. Cogn. Psychol..

[2] Chang D., Nesbitt K.V. Developing Gestalt-Based Design Guidelines for Multi-Sensory Displays. Proceedings of the ACM International Conference Proceeding Series.

[3] McDonald T.P., Pellegrino J.W. (1993). Psychological Perspectives on Spatial Cognition Thomas. Advances in Psychology.

[4] Tversky B. (1992). Distortions in cognitive maps. Geoforum.

[5] Tversky B. (1993). Cognitive Maps, Cognitive Collages, and spatial mental models. Proceedings of the European Conference on Spatial Information Theory.

[6] Tversky B. (1981). Distortions in memory for maps. Cogn. Psychol..

[7] Lopez A., Caffò A.O., Bosco A. (2021). The impact of age and familiarity with the environment on categorical and coordinate spatial relation representations. Scand. J. Psychol..

[8] Stevens A., Coupe P. (1978). Distortions in judged spatial relations. Cogn. Psychol..

[9] Lopez A., Germani A., Tinella L., Caffò A.O., Postma A., Bosco A. (2021). The road more travelled: The differential effects of spatial experience in young and elderly participants. Int. J. Environ. Res..

[10] Maki R.H. (1981). Categorization and distance effects with spatial linear orders. J. Exp. Psychol. Learn. Mem. Cogn..

[11] McNamara T.P., Hardy J.K., Hirtle S.C. (1989). Subjective hierarchies in spatial memory. J. Exp. Psychol. Learn. Mem. Cogn..

[12] Holyoak K.J., Mah W.A. (1982). Cognitive reference points in judgments of symbolic magnitude. Cogn. Psychol..

[13] Lloyd R., Heivly C. (1987). Systematic distortions in urban cognitive maps. Ann. Assoc. Am. Geogr..

[14] Kosslyn S.M., Pick H.L., Fariello G.R. (1974). Cognitive maps in children and men. Child. Dev..

[15] Newcombe N., Liben L.S. (1982). Barrier effects in the cognitive maps of children and adults. J. Exp. Child. Psychol..

[16] Hirtle S.C., Jonides J. (1985). Evidence of hierarchies in cognitive maps. Mem. Cogn..

[17] Canter D., Tagg S.K. (1975). Distance estimation in cities. Environ. Behav..

[18] Carbon C.C., Leder H. (2005). The Wall inside the brain: Overestimation of distances crossing the former Iron Curtain. Psychon. Bull. Rev..

[19] Briggs R. (1973). Urban cognitive distance. Image and Environment: Cognitive Mapping and Spatial Behavior.

[20] Allen G.L. (1981). A developmental perspective on the effects of “subdividing” macrospatial experience. J. Exp. Psychol. Hum. Learn..

[21] Byrne R.W. (1979). Memory for urban geography. Q. J. Exp. Psychol..

[22] Sadalla E.K., Staplin L.J. (1989). The perception of traversed distance, interactions. Environ. Behav..

[23] Allen G.L., Kirasic K.C. (1985). Effects of the cognitive organization of route knowledge on judgments of macrospatial distance. Mem. Cogn..

[24] Thorndyke P.W. (1981). Distance estimation from cognitive maps. Cogn. Psychol..

[25] Muller J.C. (1985). Mental maps at a global scale. Cartogr. J..

[26] Novick L.R., Tversky B. (1987). Cognitive constraints on ordering operations: The case of geometric analogies. J. Exp. Psychol. Gen..

[27] Howard J.H., Kerst S.M. (1981). Memory and perception of cartographic information for familiar and unfamiliar environments. Hum. Factors.

[28] Lloyd R. (1989). Cognitive maps: Encoding and decoding information. Ann. Assoc. Am. Geogr..

[29] Moar I., Bower G.H. (1983). Inconsistency in spatial knowledge. Mem. Cogn..

[30] Friedman A., Brown N.R. (2000). Reasoning about geography. J. Exp. Psychol. Gen..

[31] Huttenlocher J., Hedges L.V., Vevea J.L. (2000). Why do categories affect stimulus judgment?. J. Exp. Psychol. Gen..

[32] Brown C., Brandimonte M.A., Wickham L.H., Bosco A., Schooler J.W. (2014). When do words hurt? A multiprocess view of the effects of verbalization on visual memory. J. Exp. Psychol. Learn. Mem. Cogn..

[33] Fiore S.M., Schooler J.W. (2002). How did you get here from there? Verbal overshadowing of spatial mental models. Appl. Cogn. Psychol..

[34] Schooler J.W., Engstler-Schooler T.Y. (1990). Verbal overshadowing of visual memories: Some things are better left unsaid. Cogn. Psychol..

[35] Brandimonte M.A., Hitch G.J., Bishop D.V. (1992). Verbal recoding of visual stimuli impairs mental image transformations. Mem. Cogn..

[36] Brandimonte M.A., Schooler J.W., Gabbino P. (1997). Attenuating verbal overshadowing through color retrieval cues. J. Exp. Psychol. Learn. Mem. Cogn..

[37] Meilinger T., Bülthoff H.H. (2013). Verbal shadowing and visual interference in spatial memory. PLoS ONE.

[38] Hart R.A., Moore G.T. (1973). The Development of Spatial Cognition: A Review.

[39] Wang J., Schwering A. (2015). Invariant spatial information in sketch maps—A study of survey sketch maps of urban areas. J. Spat. Inf. Sci..

[40] Blades M. (1990). The reliability of data collected from sketch maps. J. Environ. Psychol..

[41] Lopez A., Caffò A.O., Postma A., Bosco A. (2020). How to separate coordinate and categorical spatial relation components in integrated spatial representations: A new methodology for analysing sketch maps. Scand. J. Psychol..

[42] Lopez A., Caffò A.O., Tinella L., Postma A., Bosco A. (2020). Studying individual differences in spatial cognition through differential item functioning analysis. Brain Sci..

[43] Fenollar P., Román S., Cuestas P.J. (2007). University students’ academic performance: An integrative conceptual framework and empirical analysis. Br. J. Educ. Psychol..

[44] Mangels J.A., Butterfield B., Lamb J., Good C., Dweck C.S. (2006). Why do beliefs about intelligence influence learning success? A social cognitive neuroscience model. Soc. Cogn. Affect. Neurosci..

[45] Richardson K., Norgate S.H. (2015). Does IQ really predict job performance?. Appl. Dev. Sci..

[46] Bosco A., Caffò A.O., Spano G., Lopez A. (2020). Beyond the cutoffs: A Bayesian approach to the use of the Montreal cognitive assessment as a screening tool for mild cognitive impairment and dementia. Diagnosis and Management in Dementia.

[47] De Goede M., Postma A. (2015). Learning your way in a city: Experience and gender differences in configurational knowledge of one’s environment. Front. Psychol..

[48] Rice M.E., Harris G.T. (2005). Comparing effect sizes in follow-up studies: ROC Area, Cohen’s d, and r. Law Hum. Behav..

[49] Ekman G., Bratfisch O. (1965). Subjective distance and emotional involvement. A psychological mechanism. Acta Psychol..

[50] Kuipers B. (1982). The “map in the head2010064 metaphor. Environ. Behav..

[51] Machielsen W.C., Rombouts S.A., Barkhof F., Scheltens P., Witter M.P. (2000). FMRI of visual encoding: Reproducibility of activation. Hum. Brain Mapp..

[52] Snyder J.P., Maling D.H. (1993). Flattening the Earth.

[53] Wen W., Kawabata H. (2018). Impact of Navon-Induced Global and Local Processing Biases on the Acquisition of Spatial Knowledge. SAGE Open.

[54] Khosla G., Rajpal N., Singh J. Evaluation of Euclidean and Manhanttan metrics in content based image retrieval system. Proceedings of the 2015 2nd International Conference on Computing for Sustainable Global Development, INDIACom.

